# Local and global patterns of admixture and population structure in Iranian native cattle

**DOI:** 10.1186/s12863-016-0416-z

**Published:** 2016-07-15

**Authors:** Karim Karimi, Eva M. Strucken, Nasir Moghaddar, Mohammad H. Ferdosi, Ali Esmailizadeh, Cedric Gondro

**Affiliations:** Department of Animal Science, Faculty of Agriculture, Shahid Bahonar University of Kerman, Kerman, PB 76169-133 Iran; School of Environmental and Rural Science, University of New England, Armidale, 2351 NSW Australia; Animal Genetics and Breeding Unit, Armidale, 2351 NSW Australia; State Key Laboratory of Genetic Resources and Evolution, Yunnan Laboratory of Molecular Biology of Domestic Animals, Kunming Institute of Zoology, Chinese Academy of Sciences, Kunming, 650223 China

**Keywords:** Admixture, Diversity, *Bos taurus*, *Bos indicus*, Domestication, Crossbreeding, World-wide, Fertile crescent

## Abstract

**Background:**

Two separate domestication events gave rise to humped zebu cattle in India and humpless taurine cattle in the Fertile Crescent of the Near and Middle East. Iran covers the Eastern side of the Fertile Crescent and exhibits a variety of native cattle breeds, however, only little is known about the admixture patterns of Iranian cattle and their contribution to the formation of modern cattle breeds.

**Results:**

Genome-wide data (700 k chip) of eight Iranian cattle breeds (Sarabi *N* = 19, Kurdi *N* = 7, Taleshi *N* = 7, Mazandarani *N* = 10, Najdi *N* = 7, Pars *N* = 7, Kermani *N* = 9, and Sistani *N* = 9) were collected from across Iran. For a local assessment, taurine (Holstein and Jersey) and indicine (Brahman) outgroup samples were used. For the global perspective, 134 world-wide cattle breeds were included. Between breed variation amongst Iranian cattle explained 60 % (*p* < 0.001) of the total molecular variation and 82.88 % (*p* < 0.001) when outgroups were included. Several migration edges were observed within the Iranian cattle breeds. The highest indicine proportion was found in Sistani. All Iranian breeds with higher indicine ancestry were more admixed with a complex migration pattern. Nineteen founder populations most accurately explained the admixture of 44 selected representative cattle breeds (standard error 0.4617). Low levels of African ancestry were identified in Iranian cattle breeds (on average 7.5 %); however, the signal did not persist through all analyses. Admixture and migration analyses revealed minimal introgression from Iranian cattle into other taurine cattle (Holstein, Hanwoo, Anatolian breeds).

**Conclusion:**

The eight Iranian cattle breeds feature a discrete genetic composition which should be considered in conservation programs aimed at preserving unique species and genetic diversity. Despite a complex admixture pattern among Iranian cattle breeds, there was no strong introgression from other world-wide cattle breeds into Iranian cattle and vice versa. Considering Iran’s central location of cattle domestication, Iranian cattle might represent a local domestication event that remained contained and did not contribute to the formation of modern breeds, or genetics of the ancestral population that gave rise to modern cattle is too diluted to be linked directly to any current cattle breeds.

**Electronic supplementary material:**

The online version of this article (doi:10.1186/s12863-016-0416-z) contains supplementary material, which is available to authorized users.

## Background

The general consensus about the origin of domesticated cattle is that two separate domestication events took place and gave rise to the variety of cattle breeds we see nowadays [1]. India is the origin of humped zebu cattle (*Bos indicus*) [2], and the Fertile Crescent of the Near East is the region of origin of humpless taurine cattle (*Bos taurus*) [3]. Iran covers the Eastern side of the Fertile Crescent bordering to the West with Turkey and Saudi Arabia, which link to Europe and Africa, and to the East to Afghanistan and Pakistan, which links to India. First agricultural remnants date back 10,000 years when also first cattle domestication is believed to have started [1, 4]. Iran is home to a variety of cattle breeds, however, only little is known about the genetic diversity of Iranian native cattle.

Globalization of breeding programs has become more important and maintaining of local genetic resources is required to facilitate rapid adaptation to changing environment. Indigenous breeds have developed unique characteristics as a response to environmental pressures such as disease and parasite tolerance, heat tolerance, and adaptation to local feed resources [5, 6]. The loss of these breeds or their genetic diversity, which is the ultimate source for the ability to adapt to a changing environment, will significantly limit future breeding programs [7]. Some of the Iranian breeds, such as Golpaigani, have already become extinct while other indigenous breeds have been shown to be on the brink of losing genetic diversity due to small effective population sizes and inbreeding [8, 9]. Middle Eastern cattle breeds represent the main links to the ancient history of taurine domestication and may also be relevant as a future source of currently untapped genetic material. Characterization of the genetic variability and breed composition of these populations will assist in guiding preservation programs and may provide additional insights into the domestication process of taurine cattle.

The availability of high density genome-wide SNP arrays has given researchers a powerful tool to characterize genetic diversity and breed composition [10–13]. Genome-wide information provides a fine-grained raster, compared to for example microsatellites, to trace even small differences between animal populations. Thus, the history of migration and mating events should be possible to be reconstructed more accurately [14].

We used high-density SNP data to investigate genetic diversity, admixture and population structures in eight Iranian native cattle breeds. Further, we incorporated our data set with information from 134 world-wide cattle breeds [10] based on 18,892 common SNPs in order to achieve an assessment of the genetic history and structure of Iranian cattle populations. These results are the first comprehensive evaluation of the valuable resource on native Iranian cattle diversity in a historically important geographic region for cattle domestication.

## Results and discussion

### Genetic structure within Iranian cattle breeds

The first part of our study was based on eight Iranian cattle breeds and three outgroups breeds (Holstein, Jersey, and Brahman sourced from the Bovine HapMap Consortium) as described in Table 1. Based on phenotypic appearances, our initial assumption was that Sistani, Mazandarani, Taleshi, and Najdi could be classified as indicine breeds; while Sarabi, Kurdi, Pars and Kermani had more similarities to taurine breeds. However, phenotypic characteristics can be misleading and for conservation purposes it is important to know whether the populations truly belong to different breeds or are just variants of the same breed [15]. Based on an analysis of molecular variances of 283,028 SNPs, we observed that the eight Iranian breeds used in this work differed significantly between each other. Breed differences accounted for 60 % of the total molecular variance (*P* < 0.001, Table 1). Conservation programs should manage the breeds individually and minimize outcrossing with foreign breeds [16] to ensure maintenance of the distinct genetic constitutions of each breed – which are already showing evidence of erosion [8, 9].

**Table 1:** Analysis of molecular variance in 8 Iranian cattle populations and 3 outgroup cattle breeds

	Source of variance	SSD	MSD	DF	F-value	Variance components (%)
Iran	Between populations	0.925	0.132	7	5.5***	60.00
	Within populations	1.612	0.024	67	-	40.00
	Total	2.537	0.034	74		100
Iran + Outgroups	Between populations	3.260	0.326	10	15.52***	82.88
	Within populations	2.288	0.021	109	-	17.12
	Total	5.548	0.047	119		100

*SSD* sums of squared deviation, *MSD* mean squared deviation, *DF* degrees of freedom

***p < 0.001: significant levels after 1000 permutations

Based on heterozygosities and inbreeding coefficients, the Kurdi and Sarabi breeds from the North-West of the country seem to have the highest genetic diversities (Table 2). This higher level of genetic variation might be due to their location close to the borders to Turkey, Armenia and Azerbaijan which allows for greater gene flow between countries. However, it is more likely that this is simply an artefact due to ascertainment bias in the design of the SNP chip [17]. Markers that have a high variability in the base population might have higher or lower variability in their allele frequencies of the study populations (ascertainment bias). Generally, it was observed that the Illumina SNP chips have a high fraction of markers that are fixed in indicine breeds which leads to the observation of less genetic diversity compared to taurine breeds. In our study there is a gradient of loss of genetic diversity as indicine breed proportions increase (Table 2). The loss of genetic diversity was assessed by overall heterozygosity, and the indicine breed proportion was calculated from Brahman breed content within a breed using ADMIXTURE [18], described later. The Sistani breed located in the South-Eastern region of the country had the lowest estimate of genetic diversity but it is also the breed with the highest level of indicine background (Table 2). This finding also lets us assume that there is more genetic diversity present in the Iranian cattle breeds than is observed based on the Illumina chip.

**Table 2:** Population genetic estimates of 8 Iranian and 3 outgroup cattle breeds based on autosomal chromosomes

Breed	N	N_e [_ 9_]_	% SNPs MAF ≤ 0.01	Average MAF	Obs. He	F_IS_	% indicine
Jersey	15	NA	14.47	0.22	0.32	−0.041	0.00001
Holstein	15	NA	8.54	0.24	0.36	−0.109	2.5
Sarabi	19	13	6.12	0.25	0.34	−0.023	42.1
Kurdi	7	29	8.52	0.26	0.36	0.005	32.3
Taleshi	7	22	15.26	0.23	0.32	0.017	61.5
Mazandarani	10	107	10.35	0.23	0.32	0.018	65.6
Najdi	7	22	15.96	0.21	0.31	−0.001	75.1
Pars	7	45	16.76	0.21	0.26	0.121	80.9
Kermani	9	23	12.01	0.20	0.27	0.083	82.6
Sistani	9	17	22.99	0.16	0.23	0.059	95.3
Brahman	15	NA	23.34	0.16	0.23	0.004	99.9

*N* sample size, *N*_*e*_ effective population size, *MAF* minor allele frequency, *He* heterozygosity, *F*_*IS*_ inbreeding coefficient, %indicine refers to the Brahman breed proportion in an ADMIXTURE analysis with K = 2

**Fig. 1:**
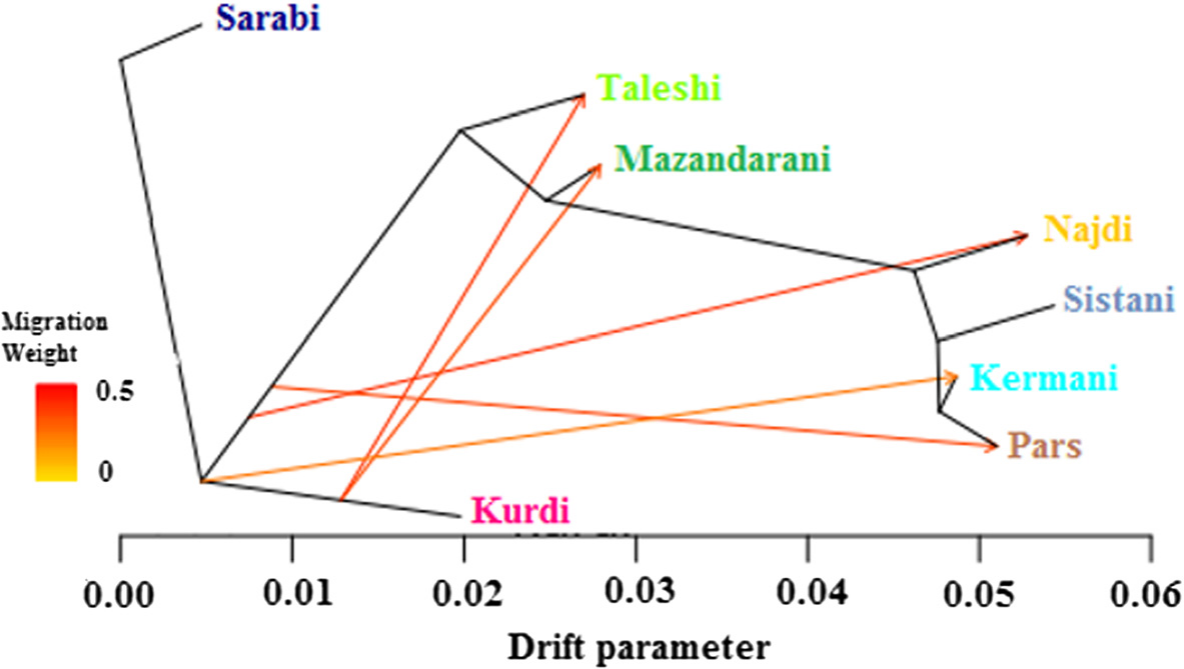
Maximum likelihood tree inferred from 8 cattle populations with five migration edges and Sarabi fixed as the root (99.89 % of variance explained). The scale bar (drift parameter) is ten times the average standard error of the sample covariance matrix between populations based on ancestral allele frequencies [19]

**Fig. 2:**
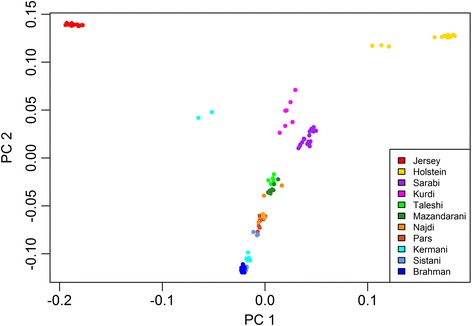
Principal components analysis of 8 Iranian cattle populations based on autosomal SNPs. Holstein, Jersey and Brahman are included as outgroups

Based on F_ST_ values, maximum likelihood phylogeny, and principal component analysis (PCA), the Sarabi breed is closest related to the Kurdi breed, both of which are located in the cool mountain area of the North-West (Table 3, Figs. 1 and 2). Separated by the Alborz mountain range and located in a temperate humid climate by the Caspian Sea are the closely related Taleshi and Mazandarani cattle (Table 3, Figs. 1 and 2). We used TreeMix [19] to analyze migration edges (migration events based on fractions of alleles passed on from an ancestral population to the descendent population), and found that five significant migration edges explained 99.8 % of the variance in the ancestry of the tree. Migration from an ancestral population of the Kurdi breed to the Taleshi and Mazandarani populations were indicated which both seem to have happened around the same time (Fig. 1).

**Table 3:** Pairwise F_ST_ values among 8 Iranian cattle breeds and 3 outgroup cattle breeds

	Jersey	Holstein	Sarabi	Kurdi	Taleshi	Mazand.	Najdi	Pars	Kermani	Sistani	Brahman
Jersey	0	-	-	-	-	-	-	-	-	-	-
Holstein	0.182	0	-	-	-	-	-	-	-	-	-
Sarabi	0.199	0.167	0	-	-	-	-	-	-	-	-
Kurdi	0.16	0.129	0.076	0	-	-	-	-	-	-	-
Taleshi	0.239	0.21	0.093	0.091	0	-	-	-	-	-	-
Mazand.	0.239	0.209	0.088	0.088	0.039	0	-	-	-	-	-
Najdi	0.274	0.24	0.111	0.12	0.075	0.055	0	-	-	-	-
Pars	0.288	0.255	0.117	0.128	0.071	0.049	0.036	0	-	-	-
Kermani	0.284	0.263	0.126	0.138	0.077	0.052	0.034	0.013	0	-	-
Sistani	0.363	0.332	0.184	0.214	0.135	0.101	0.079	0.051	0.028	0	-
Brahman	0.394	0.365	0.216	0.256	0.171	0.134	0.109	0.081	0.054	0.063	0

The semiarid and arid South of the country is inhabited by the remaining four breeds. Sistani, Pars, and Najdi are linked via the Kermani breed of the central Kerman region, which is genetically the most closely related to all three breeds (Table 3). The Kermani population has some ancestry that can be traced to a population that separated from the Sarabi into Kurdi and Taleshi. The Najdi and Pars populations showed some influx from a population that was already on the verge to separating into the branch that developed into Taleshi, Mazandarani, and the southern breeds (Fig. 1).

**Fig. 3:**
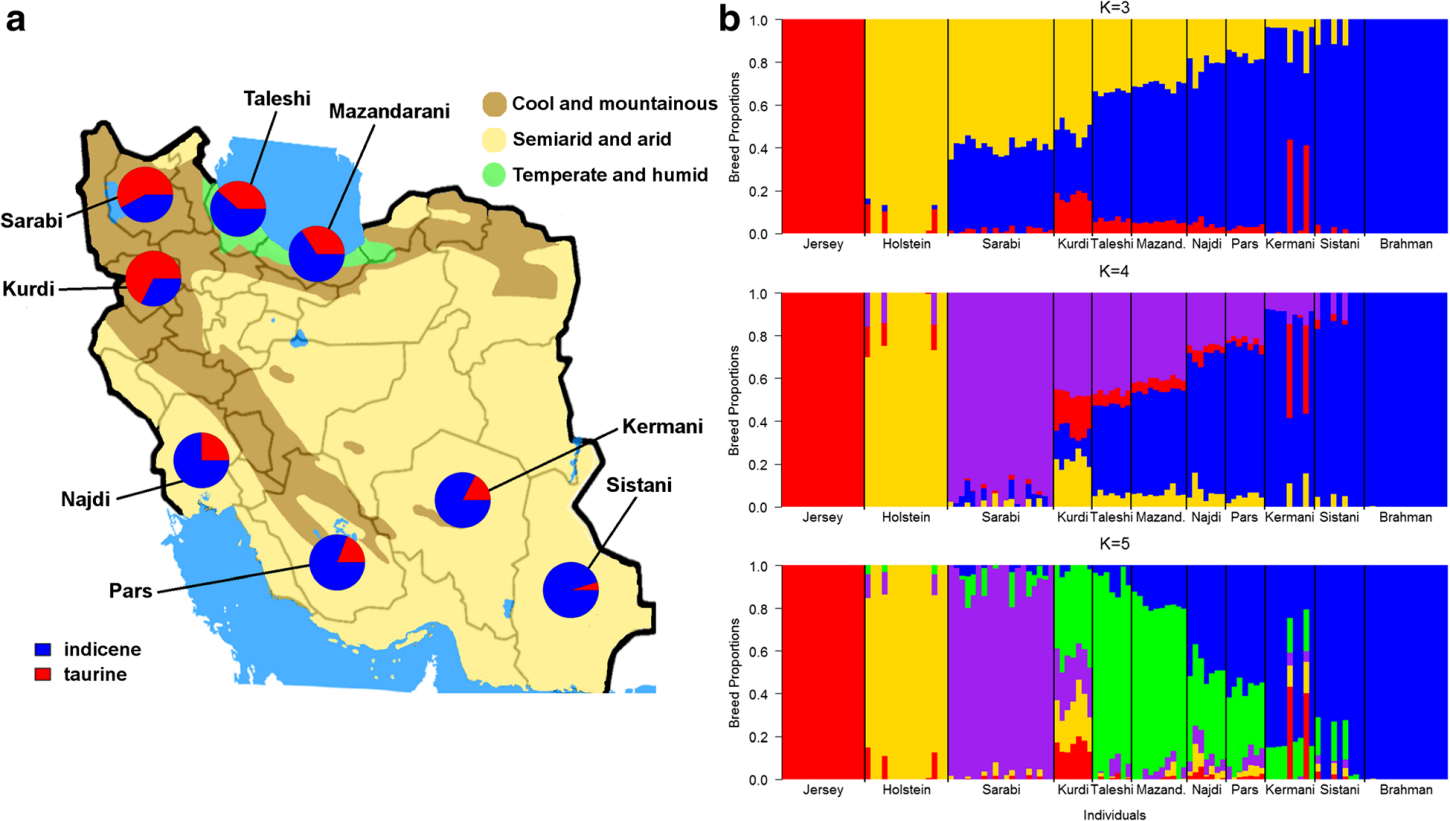
Distribution and admixture of 8 Iranian cattle populations including 3 outgroups based on 283,028 autosomal SNPs. **a** Geographic distribution of Iranian cattle breeds and their indicine and taurine proportions based on percentage of Holstein/Jersey and Brahman genetics (K = 2). **b** Breed proportions based on 3, 4, and 5 assumed founder populations in an ADMIXTURE analysis (K = 4 provided the smallest standard error of 0.557)

Finally, to infer exact breed proportions, we assumed 1 to 11 founder populations in an unsupervised ADMIXTURE analysis including Brahman as an indicine outgroup, and Holstein and Jerseys as taurine representatives. At two allowed clusters (K = 2), a clear separation between taurine and indicine backgrounds was observed (Fig. 3, Additional file 1: Figure S1). This clustering has been shown to be the dominant separator between cattle breeds [10, 20–22]. We found that the Sarabi and Kurdi populations had 57.9 and 67.7 %, respectively, attributed to taurine ancestry (Table 2). On the other side of the spectrum stands the Sistani breed with 95.3 % indicine ancestry (Table 2). As the Sistani breed is located in the South-East of the country, this indicine breed could have originated from an ancient migration directly from the Indian subcontinent which is the center of domestication of *Bos indicus* cattle [1]. The other Iranian breeds were admixed to varying degrees from these two main clusters, and could be grouped according to their geographical location (Fig. 3a). Surprisingly, the Pars and Kermani breeds showed the highest indicine breed proportion after Sistani even though we grouped them on the taurine spectrum based on their phenotype (Table 2, Additional file 1: Figure S1). This shows how misleading phenotypic breed characterization can be and potentially points out that the typical indicine breed characteristics of hump, pendulous ears, and a pronounced dewlap are controlled by only a few genetic loci or regions. Note that the presented breed proportions are only relative to the samples used in this study and should not be taken as absolute. Further, Brahman cattle are not pure indicine cattle and have around 9 % taurine background [23] which could have led to an underestimation of indicine proportions in the data.

Adding a third potential founder population separated the Holstein, Jersey, and Brahman (Fig. 3b), and revealed that the Iranian breeds are an admixture of taurine breeds, mainly corresponding to Holstein, and indicine breeds as represented by the Brahmans. To a lesser degree, a Jersey component was present in the Iranian breeds (Fig. 3b). It is often assumed that both Holstein and Jersey were used for crossbreeding with native cattle in Iran [24]. However, the low influence of Jersey in all Iranian breeds suggest that they were not widely used for crossbreeding purposes (e.g., with Najdi cattle) and the breeding history should be reconsidered.

Assuming four ancestral populations provided the lowest cross-validation standard error (0.557) indicating that this is the most likely number of ancestral breeds based on the data of this study. With four founder breeds, the Sarabi separated into its own distinct breed (an Iranian taurine). Based on word-of-mouth from local farmers, Sarabi had no introgression from other breeds for the last 50 years and represents the fourth ancestral breed of the Iranian cattle populations in our study (Fig. 3b). With a fifth ancestor population, Taleshi and Mazandarani separate from the other breeds which could be associated with their relatively isolated location by the Caspian Sea. The most admixed breed is the Kurdi, made up of a high proportion of Iranian taurine followed by a Holstein and Jersey background (Fig. 3b).

From this Iranian focused perspective, we can summarize that there is a strong West to East distribution of taurine to indicine breed proportions. Predicting a breed (taurine or indicine) based on phenotypic appearance is highly misleading and should always backed-up by genetic analyses. There has been some migration within the Iranian breeds. The Sarabi appear to be most homogenous representing an independent taurine population whilst the Sistani represent a relatively homogenous indicine population. The Kurdi were the most heterogeneous population. To anchor the Iranian breeds in a global perspective and to infer breed proportions and migration events from populations outside Iran, we included a further 134 breeds in the following section.

### Admixture patterns between Iranian cattle and other world-wide cattle breeds

**Fig. 4:**
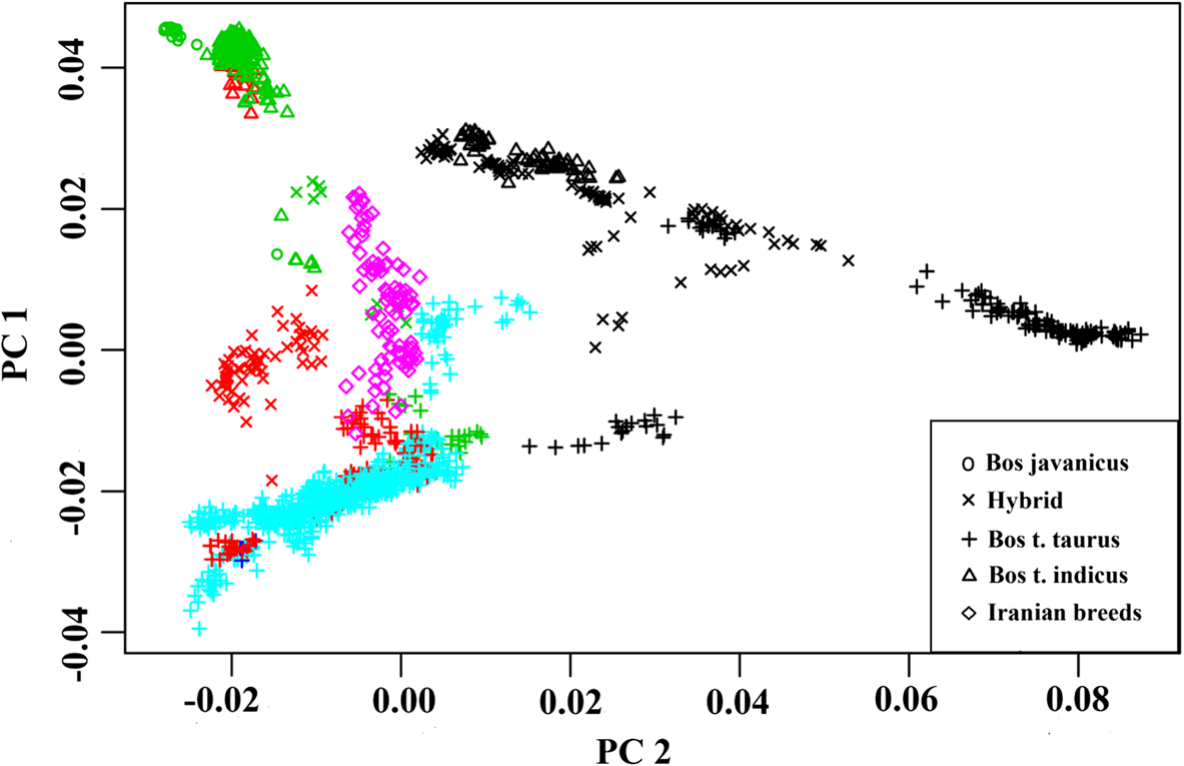
Principal components analysis of 1,632 cattle from 142 world-wide populations genotyped for 18,892 SNPs. Geographical origin of breeds are represented as: black: Africa; green: Asia; red: North and South America; cyan: Europe; and pink: Iranian

A total of 18,892 SNPs shared among 142 world-wide cattle breeds were used to anchor the Iranian cattle breeds in the global pattern of admixture (including our eight Iranian cattle breeds and 134 cattle breeds from Decker et al. 2014 [10]; Additional file 2: Table S1). The genetic relationships among breeds based on a PCA were in concordance with the geographical origin and subspecies of each breed (Fig. 4). The first PC separated indicine from taurine ancestries with the Iranian breeds spreading in between. This confirms results from the previous section, where the Iranian breeds showed different degrees of taurine to indicine ancestry following a geographical West to East distribution. The second PC separated mainly African hybrid breeds from the European, American, and Asian breeds (Fig. 4). The separation of the African hybrid breeds might be due to the presence of a unique African taurine ancestry such as the N’Dama cattle. The West African N’Dama cattle represent genetically unique taurines that are likely to be the only surviving breed directly descending from the early cattle domesticated in Africa [25, 26].

#### Admixture

We used the ADMIXTURE program in an unsupervised analysis with all 142 breeds as well as with a reduced data set of 44 breeds (highlighted breeds in Additional file 2: Table S1, note that Brown Swiss BSW were treated as two populations BRU and BSW for the analysis as per Decker et al. 2014 [10]) to infer general patterns of admixture and genetic structure. We will describe here only the analysis with 44 breeds; the detailed output with 2–5 assumed founder populations can be found in Additional files 3: Figure S2, 4: Figure S3, 5: Figure S4, and 6: Figure S5. We further calculated *f*_*3*_ statistics for all possible triplet groups of the 44 breeds, and *f*_*4*_statistics with the Iranian cattle breeds as the sister, and African taurine (Somba, Lagune, Baole, N’Dama and Oulmès Zaer), Anatolian taurine (Turkish Grey, Anatolian Black, East Anatolian Red, Anatolian Southern Yellow, and South Anatolian Red), Asian indicine (Hissar, Gabrali, Dajal, Bhagnari, Rojhan, Sahiwal, Gir, Cholistani, Tharparkar, Red Sindhi, and Achai), Asian taurine (Hanwoo, Wagyu, and Mongolian), and European taurine (Angus, Hereford, Lincoln Red, Holstein, Jersey, Guernsey, and Brown Swiss) as opposing sister groups. The *f*_*3*_ and *f*_*4*_ statistics are similar to the fixation index (F_ST_ statistic), with the exception that the genetic relationship between three or four populations are considered simultaneously rather than just between two populations. Both *f*_*3*_ and *f*_*4*_ tests were designed to detect admixture in a population based on the other populations submitted to the test.

**Fig. 5:**
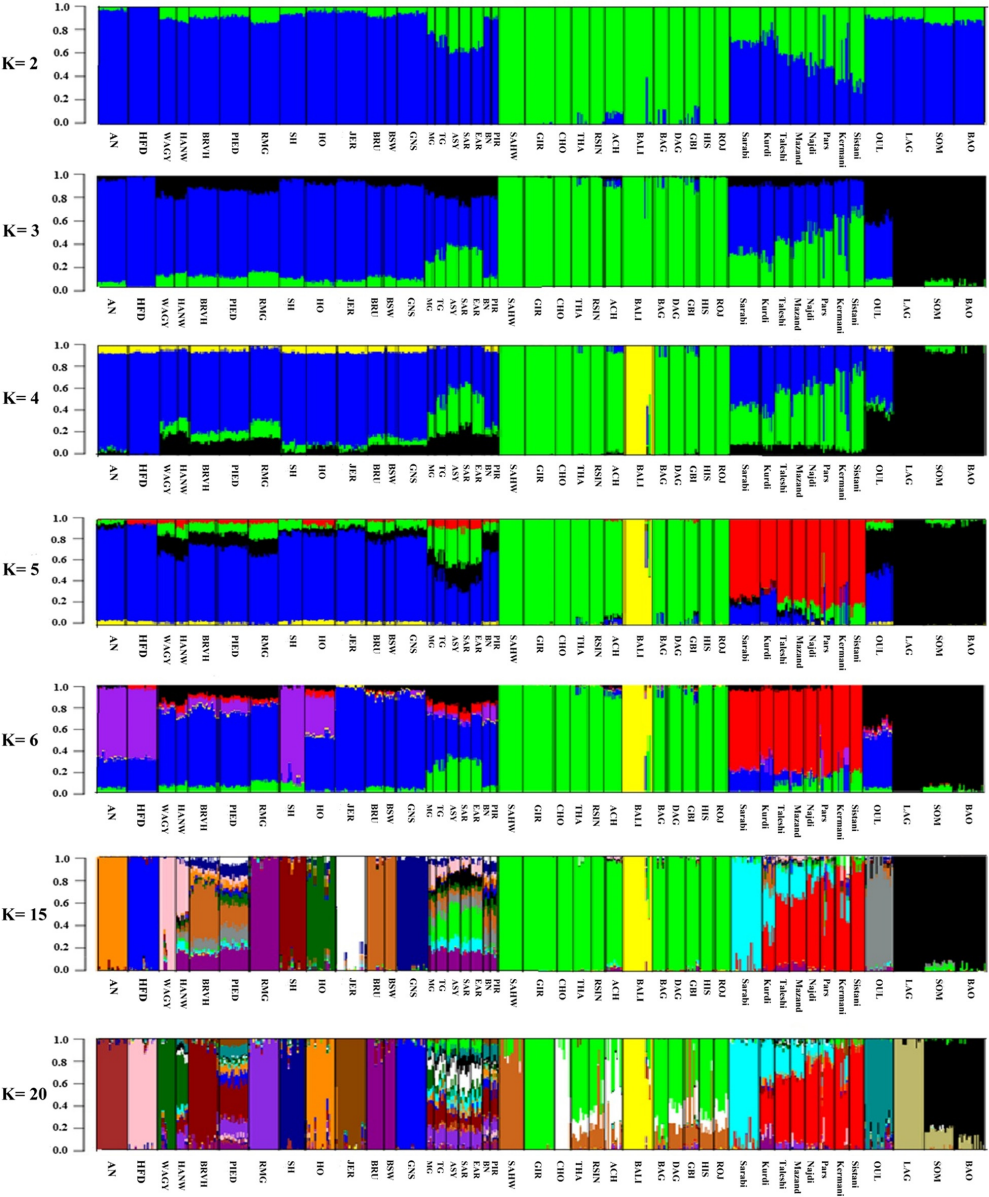
Unsupervised ADMIXTURE analysis of 584 cattle from 44 representative world-wide breeds. K = 19 provided the smallest standard error of 0.4617; breed abbreviations can be found in Additional file 2: Table S1

ADMIXTURE was run for K values from 1 to 20 with K = 19 presenting the lowest cross-validation error (0.4617). A clear separation was observed between *Bos indicus* and *Bos taurus* animals if two ancestral population were assumed (Fig. 5). The Iranian breeds showed both taurine and indicine ancestry with an East to West gradient as previously described. Estimates of indicine proportions (based on K = 2) were lower compared to the previous section. Sarabi, Kurdi, and Sistani carried now 30, 24, and 68 % indicine gene content, respectively. These lower indicine proportions are most likely due to the increased number of breeds which allow for a more detailed separation of genetic contributors.

**Fig. 6:**
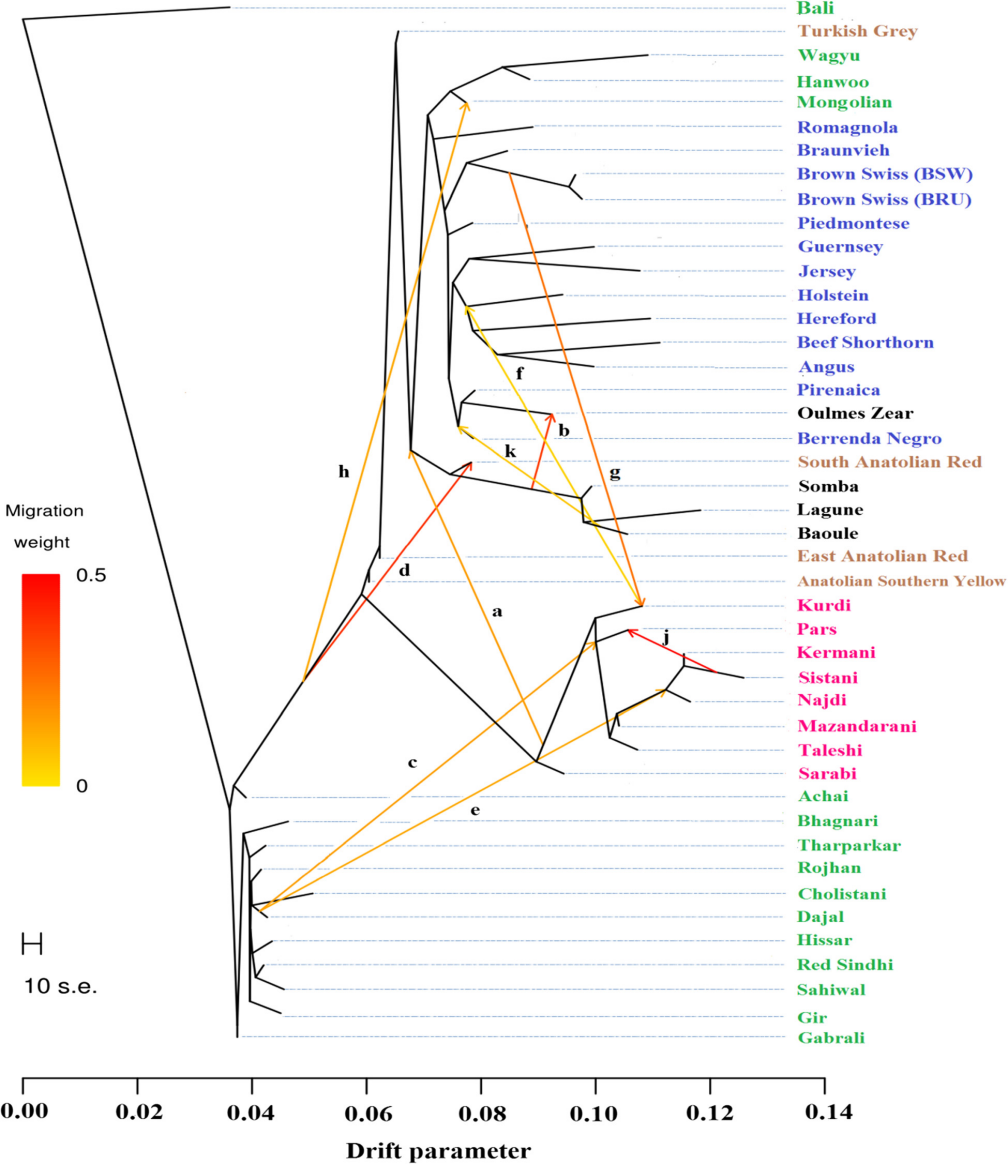
Maximum likelihood phylogenetic network inferred from 44 cattle populations with ten migration edges and Balinese cattle fixed as the root (99.32 % of variance explained). Breeds were colored according to their geographic origin; black: Africa; green: Asia; brown: Anatolian; blue: Europe; and pink: Iranian. Migration arrows are colored according to their migration weight which relates to the fraction of alleles in the descendant population that originated in the ancestral population. The scale bar (drift parameter) is ten times the average standard error of the sample covariance matrix between populations based on ancestral allele frequencies [19]

With three ancestral populations, Asian indicine, Eurasian taurine, and African taurine separated from each other. The Iranian breeds showed traces of breed proportions of African taurines potentially indicating introgression from Africa cattle (Fig. 5). On average, the north-western breeds of Mazandarani (10.2 %), Sarabi (9.85 %), Taleshi (8.7 %), and Najdi (8 %) had higher proportions of African taurine ancestry, while the south-eastern breeds of Pars (6.6 %), Kermani (5 %), and Sistani (4.1 %) showed lower African taurine introgression. Among the possible 348 tests for the *f*_*4*_ statistic, significant admixture levels were found for 62 tests. The African N’Dama and Oulmès Zaer were present in 42 and 39 significant tests, respectively, whilst only one test was significant with Lagune and Somba (*f*_*4*_ (Taleshi, Sistani; Lagune, Somba) = 0.00094, Z-score = 3.048). Nevertheless, it appears that there was no direct gene flow between African taurine and Iranian cattle breeds as no migration edges were found between these two cattle groups (Fig. 6). The traces of African taurine in the Iranian cattle might stem from a secondary source, such as foreign breed proportions within the African breeds or other breeds that carry a high proportion of African taurine ancestry. Oulmès Zaer were reported to be strongly admixed with European taurine ancestry, and introgression of indicine ancestry into N’Dama was also shown by previous studies [10, 27]. Potentially, African taurine introgression into Iranian breeds may also come from Anatolian or Mediterranean cattle breeds which both showed African taurine content. On average, African taurine ancestry was 20.8 % in Anatolian breeds, 18.4 % in Spanish breeds (Pirenaica and Berrenda en Negro), and 14.6 % Italian breeds (Romagnola and Piedmontese). Other European taurine breeds had on average 5.6 % of African taurine ancestry. Only 21 significant tests out of possible 273 tests were found for the *f*_*4*_ statistic with Anatolian breeds as opposing sister groups. Kurdi were included in 18 significant tests. The most significant test was *f*_*4*_ (Turkish Grey, Anatolian Southern Yellow; Mazandarani, Kurdi) = −0.00107 (Z-score = −6.095; alternative trees have Z-scores of 37.69 and 38.18). This result is in accordance with the geographical location of the Kurdi breed close to the border of Turkey. Iranian history was shaped by a number of invasions and conquering, such as the domination of the Assyrian Empire from the late 10th to 7th century BC, the formation of the Median Empire which included eastern Anatolia [28], and the Ottoman empire. Further, the silk road (114 BC to 1450s century AD) was a major commercial link between Europe, East Asia and Iran [29]. These events might have fostered migration of African and Anatolian cattle into Iran and vice versa, but there is no further proof or recoding of such a migration event. No significant *f*_*4*_ test was found with the Spanish breeds, however, there were five significant *f*_*4*_ tests when Italian cattle breeds (Romagnola and Piedmontese) were used as opposing sister groups. An introgression of Iranian breeds into African taurine breeds might also have occurred, however, our data did not allow for such an interpretation. Further, the African signal was lost in the Admixture analyses with K = 5 or higher and Iranian cattle breeds grouped in a distinct genetic cluster (Fig. 5).

With 19 assumed ancestral populations (lowest cross-validation error), the Sarabi formed a distinct taurine genetic cluster of which larger ancestries can be found in Kurdi, Taleshi, Mazandarani, and Pars. The Sistani as well as the Kermani formed a distinct indicine population with minor traces of Indian Gir cattle (2.1 and 3.2 %, respectively). Proportions of Sistani/Kermani were also present in large percentages in the other Iranian cattle breeds apart from Sarabi (Fig. 5). For Iranian cattle breeds, we found only 26 significant *f*_*3*_ tests that all belonged to the Kermani breed. The Sistani breed was always included as a sister group confirming the strong influence of Sistani on the formation of the Kermani breed. The most extreme test had Sistani and Jersey breeds as the sister groups with a Z-score of −9.95, which opposes our findings from the previous section in regards to the use of Jersey cattle for crossbreeding purposes with the Iranian native breeds. However, it is more likely that the entire admixture signal of the *f*_*3*_ statistic stems purely from the Sistani breed. Furthermore, *f*_*4*_ tests with Asian indicine breeds showed 43 significant tests among 420 possible tests. The most significant test included Sistani and Kurdi as sister and Achai and Gir as opposing sister groups (*f*_*4*_ = 0.0014, Z-score = 6.97). Of 43 significant tests, 35 and 11 contained Achai and Gir, respectively. Sistani had the most significant tests (26) among Iranian cattle. These results of the *f*_*4*_ statistic are in accordance with the ADMIXTURE findings of an introgression of Gir into Sistani and Kermani (Fig. 5). This introgression might be explained by the closer geographic location of these Iranian breeds to the Indian sub-continent where Gir originally stem from. The Persian Empire (550 BC-651 AD) occupied land from Africa to Eastern Europe and the Indus Valley [30]. Again, whilst migration of cattle within the Persian Empire and across its borders can historically be assumed, further proof or recordings of such a migration is lacking. No significant introgression was found with Hissar, Gabrali, Dajal, Bhagnari, or Rojhan as opposing sister groups.

As in the previous section, the Kurdi were the most heterogeneous breed and some traces of Brown Swiss were detected (max 23.3 %, min 3.4 %). Even though some Kurdi animals also showed traces of Jersey, an active crossing can still be rejected based on these results. Out of 588 *f*_*4*_ tests with European taurine cattle, 100 tests were significant, and Brown Swiss were included in most of the significant tests (46 tests), followed by Holstein (42 tests) and Jersey (27 tests).

Modern Iranian cattle breeds are located in a region associated with first cattle domestication, therefore allowing for the hypothesis that these breeds should represent some form of ancestor, we could not find substantial proof for this hypothesis. Breed proportions of Iranian populations in other world-wide cattle breeds are limited to some traces of Sarabi in Korean Hanwoo cattle, and Anatolian breeds (Mongolian, Turkish Grey, Anatolian Southern Yellow, South Anatolian Red, east Anatolian Red; Fig. 5). The geographical location of the Anatolian breeds close to Iran and the extend of historic Empires might explain migration events from Iranian breeds across borders. The breed content in the Korean Hanwoo cattle, however, might have been a larger migration such as the Silk Road traffic. Out of 85 possible *f*_*4*_ tests with Iranian cattle as sister and Wagyu, Hanwoo, and Mongolian cattle as opposing sister populations, only one significant test was found (*f*_*4*_ (Kurdi, Kermani; Hanwoo, Mongolian) = −0.0009 (Z-score = −3.55, alternative trees had Z-scores of 46.5 and 50.7).

#### Phylogeny and migration events

**Fig. 7:**
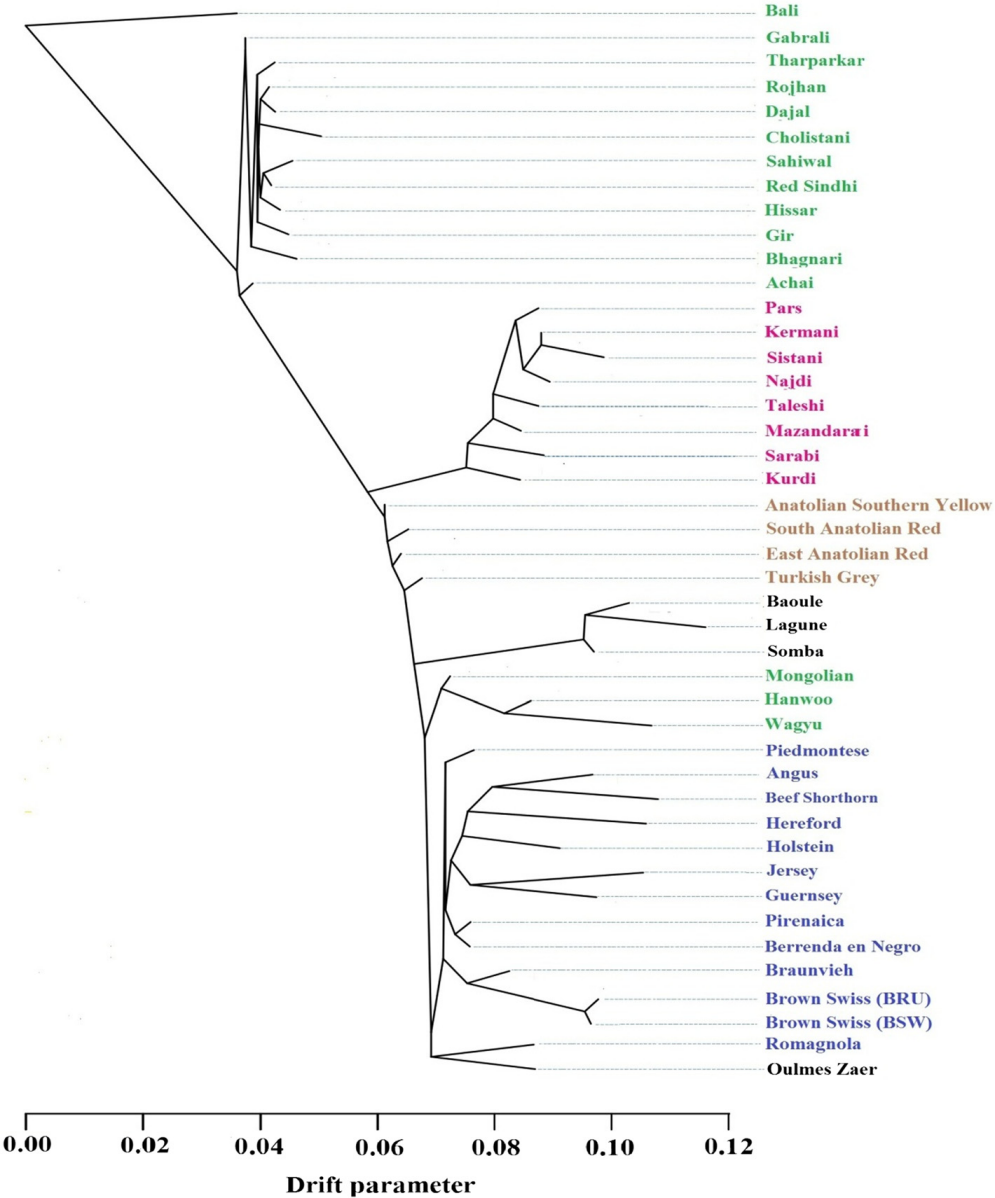
Maximum likelihood phylogenetic network inferred from 44 cattle populations and Balinese cattle fixed as the root. Breeds were colored according to their geographic origin; black: Africa; green: Asia; brown: Anatolian; blue: Europe; and pink: Iranian. The scale bar (drift parameter) is ten times the average standard error of the sample covariance matrix between populations based on ancestral allele frequencies [19]

Further investigations of phylogeny and migration events were carried out with the subset of 584 individuals from 44 breeds representing Asian indicine, Eurasian taurine, African taurine, Anatolian, and Iranian cattle breeds. At first, maximum likelihood phylogeny of the 44 breeds was created using TreeMix [19] without migration events (Fig. 7). we chose Balinese cattle as an outgroup forming the root of the tree rather than an Iranian breed to avoid any bias towards the hypothesis that Iranian breeds should be an ancestral breed based on their geographic location. The first branch that separated from the root included clusters of Asian indicine. The next major branch included the Iranian cattle breeds which remained one breed even after European breeds such as Angus, Hereford, or Holstein developed (Fig. 7). Kurdi and Sarabi separated first into distinct groups confirming results from the previous section. Iranian breeds with higher indicine content separated last. The individual branches for the Iranian breeds were relatively short compared to European taurine breeds (indicating a lesser amount of genetic diversification), but longer compared to Asian indicine, African taurine, or Anatolian breeds.

Ten migration events based on allele fractions between the populations were sequentially added with the final model explaining 99.32 % of the variance in relatedness between populations (Fig. 6). As in the ADMIXTURE and *f*_*4*_ analyses, a larger influx of Brown Swiss into the Kurdi population was observed, underpinning that there was no active crossing with Jersey cattle. The Iranian breeds with larger indicine breed proportions (Najdi, Pars, Kermani, and Sistani) were influenced by a common ancestor with indicine Dajal cattle (Punjab, Pakistan). Dajal cattle have more than 50 % of Gir content according to our ADMIXTURE analysis which might explain the Gir proportion that we previously observed in the Sistani and Kermani breeds (Fig. 5).

Several migration events of Iranian cattle breeds were observed. A strong indication is given for a crossing of Sistani with Pars cattle, which was not observed in the previous section (Fig. 6). Further, migration from the Kurdi population into an ancestral population of the European Holstein, as well as from a common ancestor of the Kurdi to an ancestral population of African cattle breeds (Somba, Lagune, and Baoule) was observed and in concordance with our *f*_*4*_ statistics. The Sarabi breed appears to be excluded from migration events confirming their unique position within the Iranian breeds.

## Conclusion

Our results provide novel information about the genetic structure and admixture of present day cattle breeds inhabiting a center of a historical domestication event. We showed that the eight Iranian cattle breeds feature discrete genetic characteristics which have to be considered in conservation programs aiming at preserving unique species and genetic diversity. Despite a complex admixture pattern among Iranian cattle breeds, we did not find strong introgression from other world-wide cattle breeds into Iranian cattle. A clear geographical distribution of taurine influences in the North-West and indicine influence in the South-East was found with Sarabi forming a unique taurine breed and Sistani and Kermani forming unique indicine breeds. Other Iranian breeds are, with minor exceptions, composed of these unique Iranian breeds. Minor introgression from Romagnola, Brown Swiss, and Gir were found, however, in such low quantities that a prolonged or breed changing admixture could be excluded. Further, minor contents of the Iranian Sarabi breed were found in Korean Hanwoo and Anatolian cattle breeds. Despite the geographic location of the Iranian breeds in an ancient centre of cattle domestication, we did not find more evidence of introgression of these breeds into other world-wide cattle breeds. Possible interpretations of these results are: 1. Iranian breeds remained fairly unchanged since their formation and main migration events occurred within the country. Thus, the Iranian cattle breeds might be seen as a living link to ancient domestication events; however, their contribution to the formation of modern cattle breeds seems to be marginal. Whilst there might have been only two major domestication events leading to the differentiation of *Bos taurus* and *Bos indicus* breeds, many smaller and local events might have taken place at the same time, with the Iranian breeds representing one of these local domestication events that survived into the present. 2. Historical invasions and migration routes between Europe, Africa, and Asia resulted in a highly admixed cattle population inhabiting the ancient Fertile Crescent. Traces of this ancestral population might be so diluted in modern cattle breeds that it is difficult to pin-point the contribution or genetic link between cattle breeds of Iran and other global breeds.

## Methods

### Samples

Hair samples were collected from a total of 90 individuals representing eight different breeds of Iranian native cattle. As far as possible, unrelated animals were selected either based on pedigree recordings or information provided by farmers. To ensure a good representation of breeds across Iran, we considered characteristics of the area from which the cattle were sampled such as ethnic community, production system (pastoral or crop and livestock), and ecological zones (Fig. 3). Samples were collected from the following breeds: Sarabi (*N* = 20), Kurdi (*N* = 10), Taleshi (*N* = 10), Mazandarani (*N* = 10), Najdi (*N* = 10), Pars (*N* = 10), Kermani (*N* = 10), and Sistani (*N* = 10). We further included Holstein, Jersey, and Brahman cattle samples sourced from the Bovine HapMap Consortium to be taurine and indicine outgroups to the Iranian samples. Regarding the prominent role of Holstein and Jersey breeds in crossbreeding programs with Iranian cattle, this information was used to check whether animals correctly represented their predefined populations. In a global analysis, 1,557 animals from 134 cattle breeds sourced from Decker et al. (2014) [10] were used instead of the Bovine HapMap data.

### Genotyping and quality control

Genomic DNA of the Iranian samples was extracted from hair roots and SNP genotyping was performed using the Illumina high-density Bovine BeadChip (Illumina, Inc, San Diego, CA, USA) designed to genotype 777,962 SNPs. Quality control of the autosomal genotypes was performed using the program snpQC [31] across all Iranian breeds and per outgroup breed. Filtering parameters included a GC score >0.9 (418,571 SNPs failed), call rates per marker >90 % (295,607 SNPs failed) and per animal >70 % (14 animals failed). Further, markers that deviated in their heterozygosity by more than 3 standard deviations from the mean heterozygosity (3,243 SNPs failed), and that deviated from Hardy-Weinberg equilibrium at P-value < 10e–16 (7,550 SNPs failed) were excluded. After quality control, 283,028 SNPs that passed the filter criteria in all breeds remained for further analysis. The number of Iranian samples used after quality control was 19 Sarabi, 7 Kurdi, 7 Taleshi, 10 Mazandarani, 7 Najdi, 7 Pars, 9 Kermani, and 9 Sistani animals. From the quality controlled outgroup breeds, 15 animals per breed were selected to create a balanced data set.

### Genetic differentiation and population structure within Iranian cattle breeds

Inbreeding coefficients (F_IS_) and pairwise F_ST_ values, which describe the genetic differentiation between two populations, were estimated according to Weir and Cockerham (1984) [32]. Analysis of molecular variances (AMOVA) [33] were carried out with the StAMPP package in R [34]. The AMOVA analysis provides further information on the genetic differentiation within a population and between populations. Further, a principal components analysis based on the genetic data (PCA) was carried out with the SNPRelate package in R [35] describing patterns of population differentiation and overlap. Clustering of breeds into genetic groups and estimation of breed proportions of ancestral breeds was carried out with an unsupervised analysis in ADMIXTURE 1.23 [36]. The best number of ancestral populations (K) was inferred via the program’s cross-validation procedure for 1 to 11 assumed populations. The number of ancestral populations with the best predictive accuracy was based on the lowest standard error of the cross-validation error estimate. These analyses were carried out with three outgroup breeds (Holstein, Hersey, and Brahman) to anchor the eight Iranian cattle breeds. The indicine breed proportion was calculated as the proportion of the Brahman breed at K = 2. The TreeMix software [19] was used to model gene flow between the Iranian cattle populations and create maximum likelihood trees. TreeMix computes a covariance matrix of all populations based on allele frequencies, comparing two populations (X_i_ and X_j_) with respect to a common ancestral population (xA) (Cov (X_i_,X_j_) = E[(X_i_-xA)(X_j_-xA)]). Migration events are modelled by allowing ancestry from multiple ancestral populations and weighting the contribution of each ancestral population by its fraction of alleles in the descendent population. Four independent runs with different seeds for five migration edges were carried out and Sarabi were set as the root of the tree due to their clear separation from other breeds in the previously described analyses.

### Placement of Iranian cattle breeds in world-wide patterns of admixture

Genotyping data from Iranian cattle samples were merged with the data set from Decker et al. (2014) [10]. The final data set comprised 1,632 individuals from 142 cattle breeds genotyped for 18,892 common autosomal SNPs. As before, the SNPRelate package [35] was used for a PCA analysis.

To decrease computing time, 584 individuals from 44 cattle breeds were selected from Decker et al. (2014) [10]. Selection was based on purity (based on PCA results), number of samples, and best representation of major genetic groups: European taurine, African taurine, Asian taurine, Anatolian cattle, Iranian cattle, and indicine populations. An unsupervised ADMIXTURE analyses was run for K values from 1 to 20 with a cross-validation procedure for the complete (142 breeds) and reduced dataset (44 breeds). Maximum likelihood phylogeny of the 44 breeds was created in TreeMix [19] with and without migration events. Balinese cattle were set as the graph root and blocks of 1,000 SNPs were used to account for potential linkage disequilibrium between nearby SNPs. Ten migration edges were sequentially added. Four independent runs with different seeds were carried out to examine the consistency of the migration edges. Furthermore, the THREEPOP program implemented in TreeMix was applied to evaluate the admixture history among populations based on *f*_*3*_ [37] and *f*_*4*_ [38] statistics. The *f*_*3*_ statistic was carried out for all possible triplets among the 142 breeds [*f*_*3*_(X; A, B)]. The *f*_*4*_ statistic was calculated for several subsets of the populations using the FOURPOP program from TreeMix [*f*_*4*_(X, Y; A, B)]. Both the *f*_*3*_ and *f*_*4*_ tests are designed to detect admixture in a population based on the other populations submitted to the test.

## Abbreviations

AD, after Christ; AMOVA, analysis of molecular variance; BC, before Christ; DF, degrees of freedom; F_IS_, inbreeding coefficient; F_ST_, fixation index; GC score, GenCall score, confidence measure for genotype calling; He, heterozygosity; K, number of assumed ancestral population in an ADMIXTURE analysis; MAF, minor allele frequency; MSD, mean square error; N, sample size; NA, data not available; N_e_, effective population size; PCA, principal component analysis; SNP, single nucleotide polymorphism; SSD, sums of square deviation

## Additional files

Additional file 1: Figure S1. Breed proportions of 8 Iranian and 3 outgroup cattle breeds for 2–11 assumed founder populations in an unsupervised ADMIXTURE analysis. (K = 4 provided the smallest cross-validation error). (TIF 378 kb) Additional file 2: Table S1. Number of samples, species, subspecies assignments, and geographical provenance of each population. With the exception of 8 Iranian native cattle breeds, sample descriptions are according to Decker et al. 2014 [10]. (XLSX 17 kb) Additional file 3: Figure S2. Breed proportions based on 2 assumed founder populations in an unsupervised ADMIXTURE analysis of 142 world-wide cattle breeds. blue: Eurasian *Bos taurus* ancestry; green: *Bos javanicus* and *Bos indicus* ancestry; breed abbreviations can be found in Additional file 2: Table S1. (TIFF 689 kb) Additional file 4: Figure S3. Breed proportions based on 3 assumed founder populations in an unsupervised ADMIXTURE analysis of 142 world-wide cattle breeds. blue: Eurasian *Bos taurus* ancestry; green: *Bos javanicus* and *Bos indicus* ancestry; breed abbreviations can be found in Additional file 2: Table S1. (TIFF 608 kb) Additional file 5: Figure S4. Breed proportions based on 4 assumed founder populations in an unsupervised ADMIXTURE analysis of 142 world-wide cattle breeds. blue: Eurasian *Bos taurus* ancestry; green: *Bos javanicus* and *Bos indicus* ancestry; breed abbreviations can be found in Additional file 2: Table S1. (TIF 679 kb) Additional file 6: Figure S5. Breed proportions based on 5 assumed founder populations in an unsupervised ADMIXTURE analysis of 142 world-wide cattle breeds. blue: Eurasian *Bos taurus* ancestry; green: *Bos javanicus* and *Bos indicus* ancestry; breed abbreviations can be found in Additional file 2: Table S1. (TIFF 682 kb)
