# Supplementary figures and images for: Local and global patterns of admixture and population structure in Iranian native cattle
Source: BMC Genet. 2016 Jul 15;17:108. doi: 10.1186/s12863-016-0416-z (PMC4946207; doi:10.1186/s12863-016-0416-z)

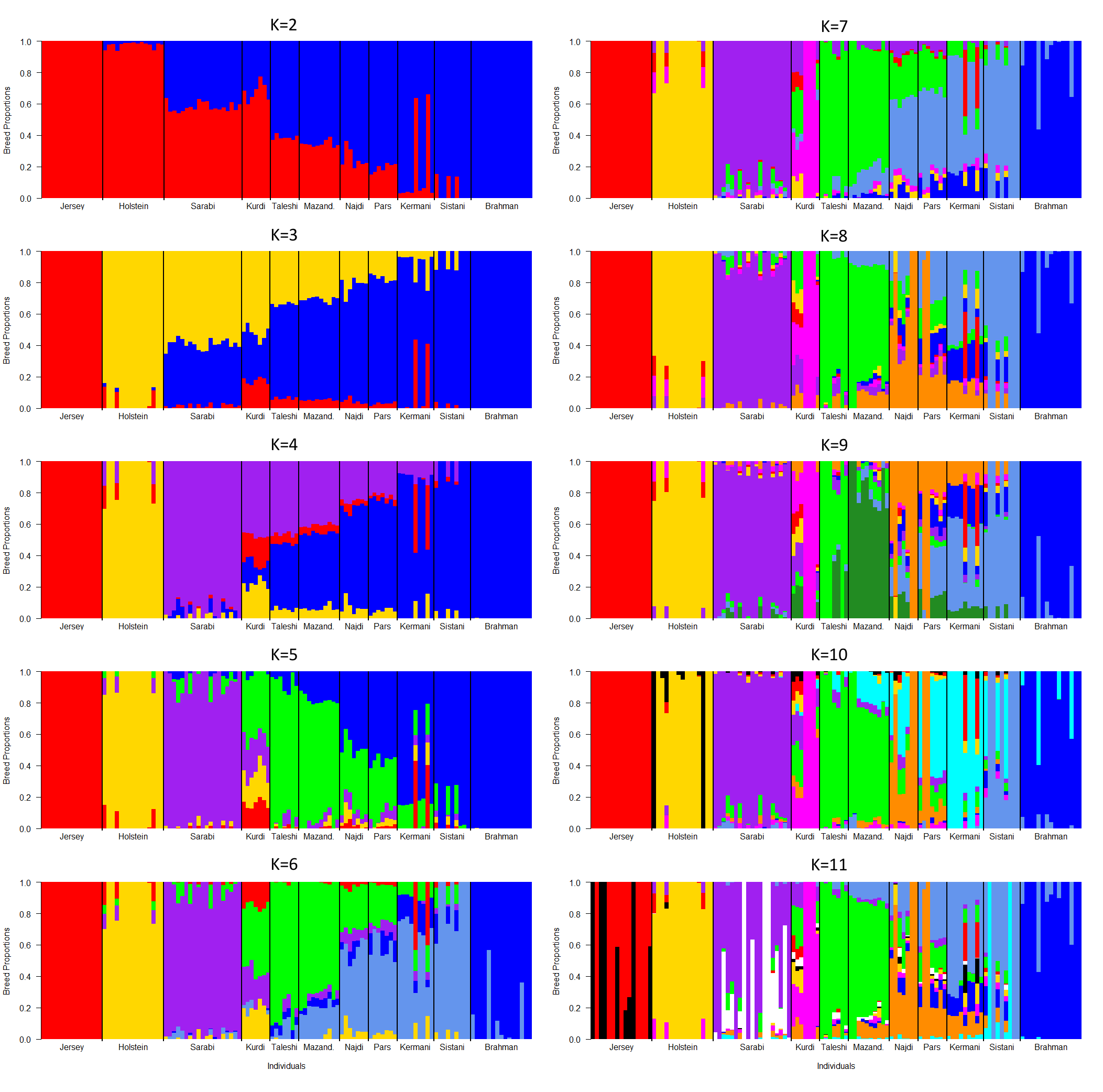

Supplement: Additional file 1: Figure S1. — Breed proportions of 8 Iranian and 3 outgroup cattle breeds for 2–11 assumed founder populations in an unsupervised ADMIXTURE analysis. (K = 4 provided the smallest cross-validation error). (TIF 378 kb) [file 12863_2016_416_MOESM1_ESM.tif]

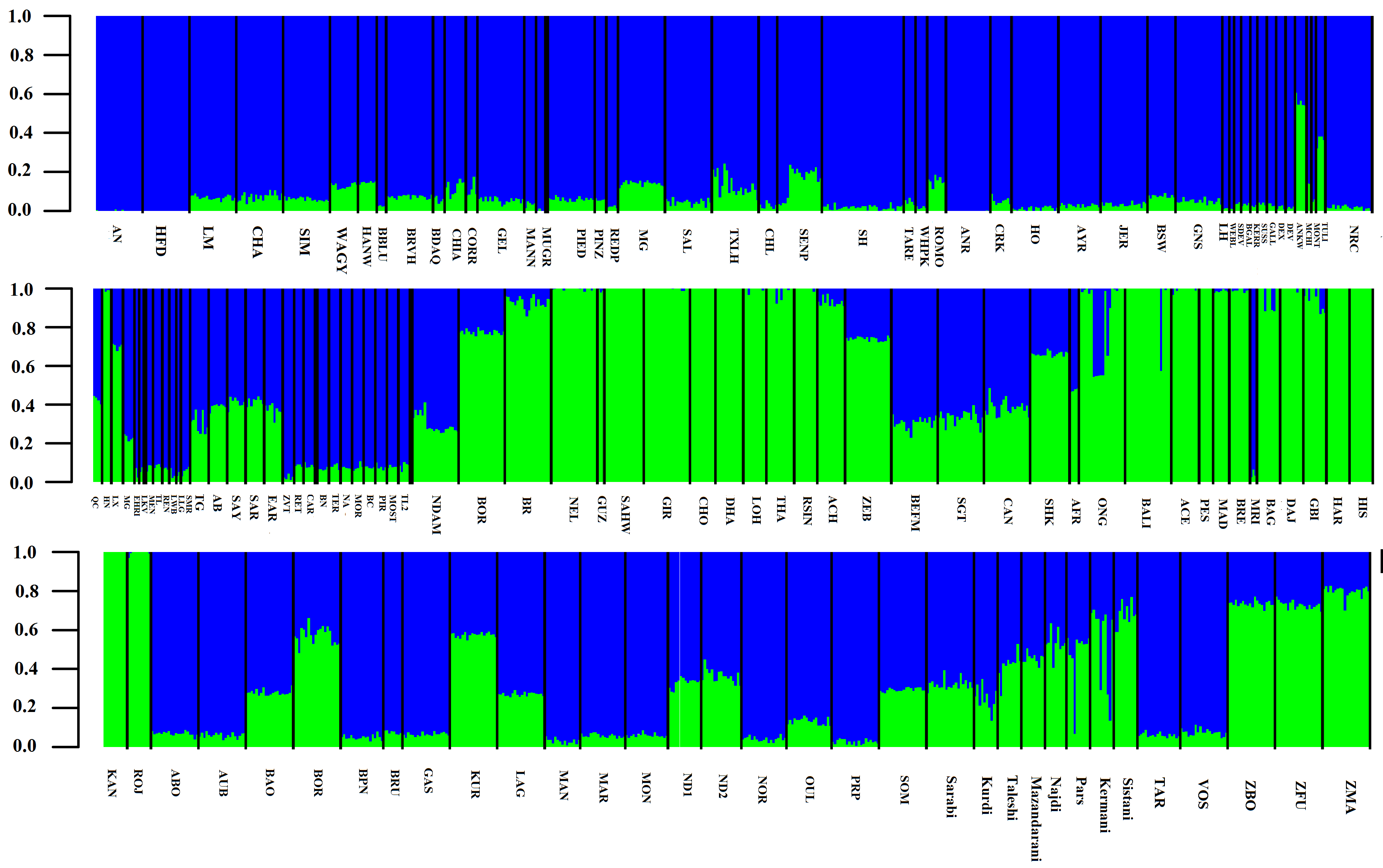

Supplement: Additional file 3: Figure S2. — Breed proportions based on 2 assumed founder populations in an unsupervised ADMIXTURE analysis of 142 world-wide cattle breeds. blue: Eurasian Bos taurus ancestry; green: Bos javanicus and Bos indicus ancestry; breed abbreviations can be found in Additional file 2: Table S1. (TIFF 689 kb) [file 12863_2016_416_MOESM3_ESM.tiff]

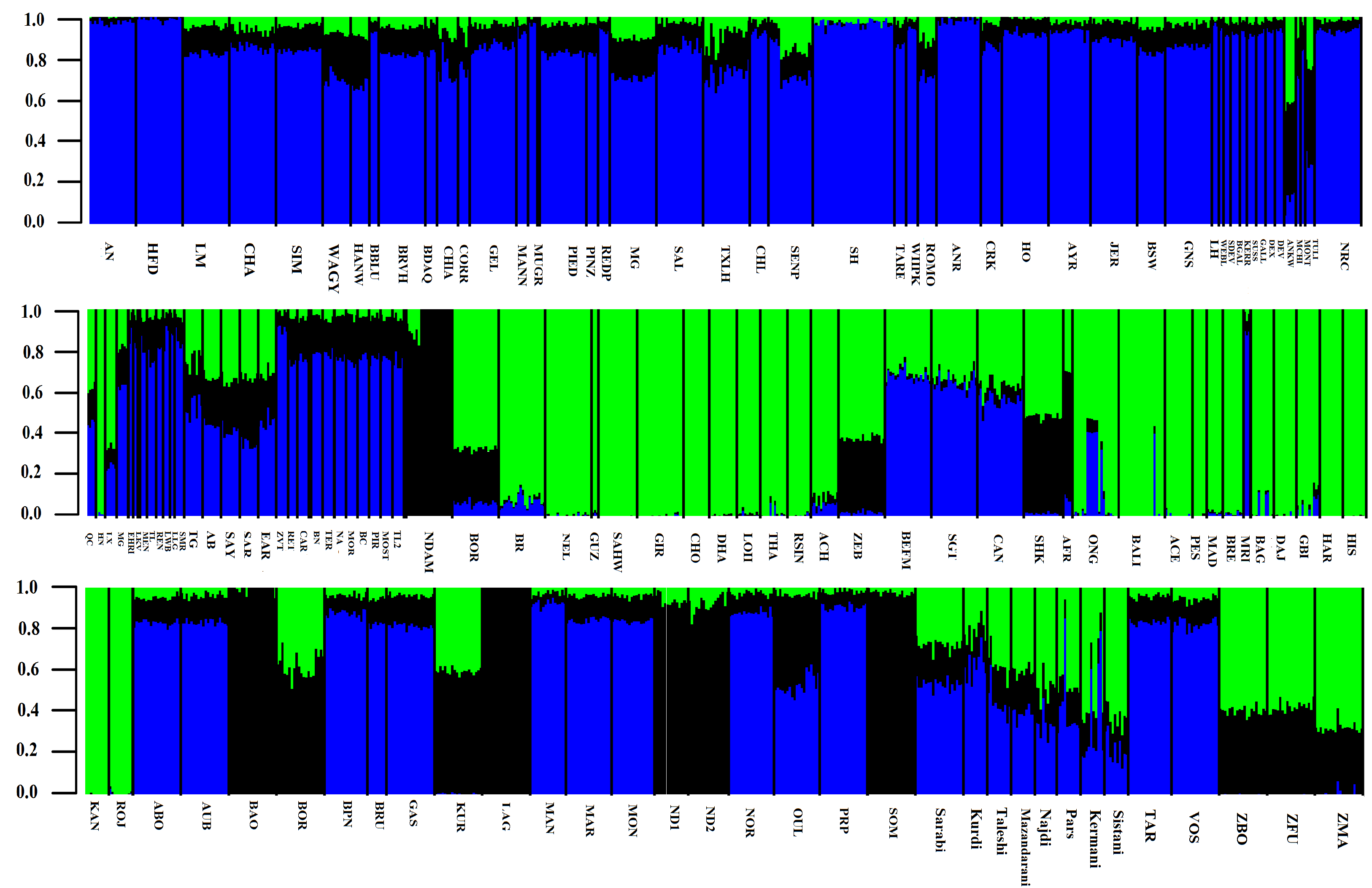

Supplement: Additional file 4: Figure S3. — Breed proportions based on 3 assumed founder populations in an unsupervised ADMIXTURE analysis of 142 world-wide cattle breeds. blue: Eurasian Bos taurus ancestry; green: Bos javanicus and Bos indicus ancestry; breed abbreviations can be found in Additional file 2: Table S1. (TIFF 608 kb) [file 12863_2016_416_MOESM4_ESM.tiff]

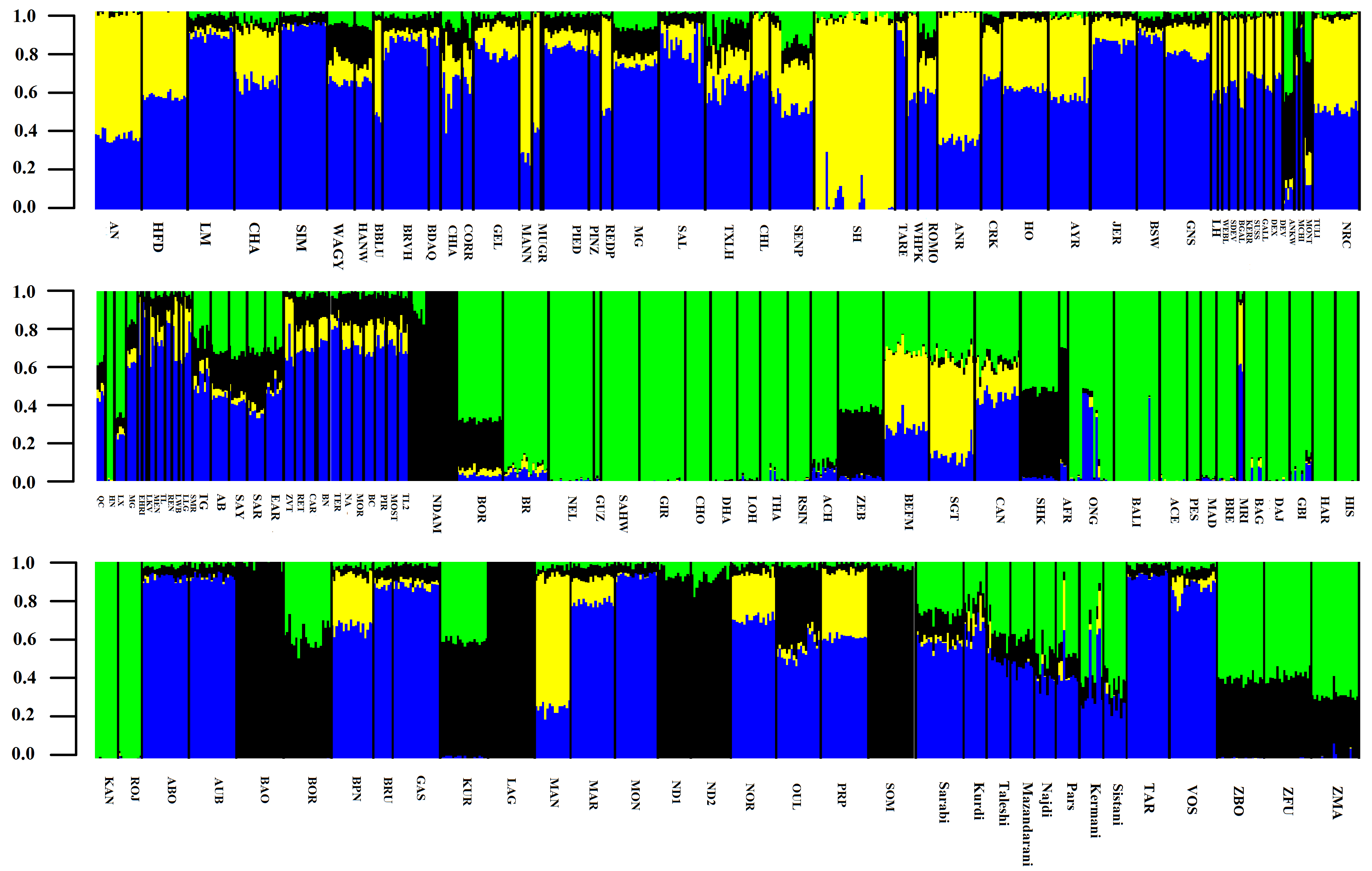

Supplement: Additional file 5: Figure S4. — Breed proportions based on 4 assumed founder populations in an unsupervised ADMIXTURE analysis of 142 world-wide cattle breeds. blue: Eurasian Bos taurus ancestry; green: Bos javanicus and Bos indicus ancestry; breed abbreviations can be found in Additional file 2: Table S1. (TIF 679 kb) [file 12863_2016_416_MOESM5_ESM.tif]

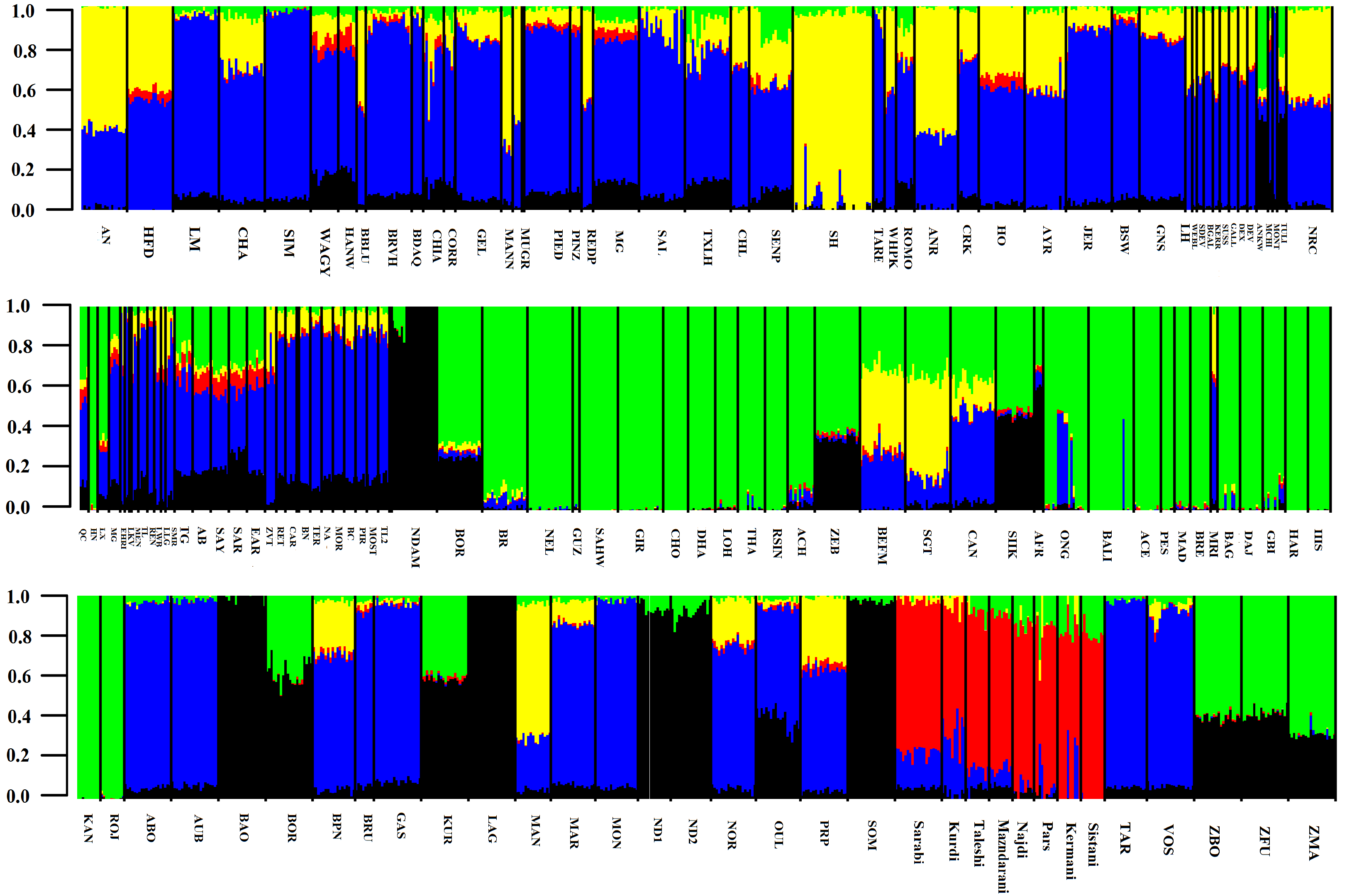

Supplement: Additional file 6: Figure S5. — Breed proportions based on 5 assumed founder populations in an unsupervised ADMIXTURE analysis of 142 world-wide cattle breeds. blue: Eurasian Bos taurus ancestry; green: Bos javanicus and Bos indicus ancestry; breed abbreviations can be found in Additional file 2: Table S1. (TIFF 682 kb) [file 12863_2016_416_MOESM6_ESM.tiff]
